# Fruit Vinegars as Natural and Bioactive Chitosan Solvents in the Production of Chitosan-Based Films

**DOI:** 10.3390/polym17010011

**Published:** 2024-12-25

**Authors:** Karolina Stefanowska, Magdalena Woźniak, Renata Dobrucka, Anna Sip, Lucyna Mrówczyńska, Agnieszka Waśkiewicz, Izabela Ratajczak

**Affiliations:** 1 Department of Chemistry, Faculty of Forestry and Wood Technology, Poznan University of Life Sciences, Wojska Polskiego 75, 60625 Poznan, Poland; karolina.stefanowska@up.poznan.pl (K.S.); agnieszka.waskiewicz@up.poznan.pl (A.W.); izabela.ratajczak@up.poznan.pl (I.R.); 2 Department of Industrial Products and Packaging Quality, Institute of Quality Science, Poznań University of Economics and Business, al. Niepodległości 10, 61875 Poznan, Poland; renata.dobrucka@ue.poznan.pl; 3 Department of Biotechnology and Food Microbiology, Faculty of Food Science and Nutrition, Poznan University of Life Sciences, Wojska Polskiego 48, 60627 Poznan, Poland; anna.sip@up.poznan.pl; 4 Department of Cell Biology, Faculty of Biology, Adam Mickiewicz University, Uniwersytetu Poznańskiego 6, 61614 Poznan, Poland; lucyna.mrowczynska@amu.edu.pl

**Keywords:** antimicrobial effect, antioxidant activity, mechanical properties

## Abstract

Natural fruit vinegars, derived from various fruits, enhance culinary experience and offer potential health benefits due to their bioactive compounds. In this study, fruit vinegars (apple, blackcurrant, and cherry) were used as natural solvents for producing chitosan films, introducing an environmentally friendly approach. Fruit vinegars and chitosan-based solutions were examined for their antioxidant and antimicrobial properties. In turn, the obtained chitosan films were characterized by their antimicrobial, mechanical, and structural properties. Both fruit vinegars and film-forming chitosan solutions showed antioxidant activity, and chitosan–cherry vinegar solutions exhibited the highest antiradical and ferrous ion-chelating effect. All solvents and chitosan-based solutions were characterized by antimicrobial properties, especially against *Pseudomonas aeruginosa* (inhibition zone > 28 mm). Antimicrobial activity was also preserved in the case of chitosan-based film, especially when produced with cherry vinegar, which showed activity against the broadest spectrum of bacteria. The largest zone of inhibition for all samples was observed for *P. aeruginosa* in the range of 19 mm from the inhibition zone to >28 mm, depending on the type of vinegar used as a solvent. The conducted tests showed that the type of vinegar used also affects the mechanical parameters of the films obtained, such as elongation at break, for which values were recorded from 3.97 to 4.93 MPa, or tensile strength, for which the values were recorded from 48.48 to 70.58 MPa. The results obtained demonstrate that natural fruit vinegars, serving as chitosan solvents, can be an alternative to traditionally used acidic solvents, yielding films with favorable properties.

## 1. Introduction

Chitosan, a biopolymer derived from chitin, holds promising prospects in the production of films and packaging materials due to its advantageous properties. As a polysaccharide, chitosan exhibits biodegradability, making it an eco-friendly alternative to conventional plastic materials [1]. Its film-forming ability, oxygen barrier properties, and antimicrobial activity make it suitable for various packaging applications, particularly in the food industry, where extending shelf life and maintaining product freshness are paramount [1,2,3]. The use of chitosan as a natural biopolymer in food packaging makes it possible to take advantage of its positive capabilities to protect food from spoilage while reducing dependence on synthetic plastics [1].

In order to increase biological activity, especially the antimicrobial and antioxidant properties of chitosan films, various bioactive additives, such as essential oils [4] or plant extracts [5], among others, are added to the chitosan matrix. Current research efforts are focused on identifying novel and bioactive solvents, thus paving the way for the development of innovative chitosan-based packaging materials with enhanced functionality and sustainability. A particularly interesting approach is the use of natural solutions, which, in addition to the ability to dissolve chitosan, also have beneficial biological properties, including kombucha solutions or vinegars [3,6]. Chitosan-based films obtained by dissolving chitosan in tea and coffee kombucha solutions showed activity against *Salmonella* Enterica and *Pseudomonas aeruginosa*, especially when a black tea kombucha solution was used as the solvent [3]. The chitosan films prepared with pomegranate and hawthorn vinegars exhibited high activity against *Staphylococcus aureus* and *Escherichia coli* [6].

The production process of natural fruit vinegars, based on carefully controlled fermentation, imparts not only a unique flavor but also an abundance of bioactive substances, which appear to positively impact human health [7]. Numerous studies indicate that fruit vinegars have many benefits when it comes to human health, positively influencing glucose and lipid control [8] while also playing a crucial role in preventing hypertension [9] with proven anticancer effects [10]. Vinegars produced from fruits are rich in bioactive compounds like amino acids, organic acids, phenols, and mineral compounds. Depending on the raw material subjected to the fermentation process and the production methods employed, fruit vinegars exhibit varying degrees of antioxidant and antimicrobial activity [11,12,13]. The predominant acid in fruit vinegars is acetic acid, which inhibits the growth of microorganisms, including *P. aeruginosa*, *E. coli*, *S. aureus*, *Listeria monocytogenes*, *Klebsiella pneumoniae*, *Enterococcus hirae*, and *Aspergillus brasiliensis* [14,15].

The purpose of this study was to obtain chitosan-based films where fruit vinegars were used as natural and bioactive solvents for chitosan. The results of Adimcilar et al. [6] indicate that natural vinegars (grape, pomegranate, hawthorn, and apple) have great potential in the production of chitosan-based films, and their biological and physico-mechanical properties suggest their potential application in the food packaging industry. In this study, three types of fruit vinegars commonly used in Polish cuisine—apple, cherry, and blackcurrant—were tested as natural and bioactive solvents for dissolving chitosan in the production of chitosan-based films. Notably, this is the first study to explore the use of blackcurrant and cherry vinegars as solvents for chitosan. The study focused on comparing the antimicrobial, structural, and mechanical properties of chitosan films made with these three types of vinegar.

## 2. Materials and Methods

### 2.1. Preparation of Chitosan Solutions and Chitosan Films

Chitosan from crab shells (viscosity > 400 mPa·s, Sigma Aldrich, Darmstadt, Germany) was used to prepare chitosan solutions and films. Chitosan (5 g) was dissolved in 500 mL of solvents—fruit vinegars. Three types of vinegar were used: apple, blackcurrant, and cherry, which were purchased from Olini (Dziećmorowice, Poland). The solutions were mixed for 2 h using a mechanical stirrer (Heidolph Instruments GmbH & KG, Schwabach, Germany). The chitosan solutions for further tests were stored at −4 °C until analysis. Chitosan films were prepared by pouring the chitosan solutions onto Petri dishes with Teflon inserts and dried at room temperature for 24 h. As a result of the experiment, three chitosan solutions and three chitosan films were obtained, for which the symbols and compositions are presented in Table 1.

### 2.2. Characteristics of Fruit Vinegars

The pH value of acetic acid and fruit vinegars was measured using a Hanna Edge HI2002-02 pH meter (Hanna Instruments, Olsztyn, Poland).

In fruit vinegars, the concentration of lactic and acetic acid was determined chromatographically. Fruit vinegars were filtered through a 0.45 μm syringe filter (Chromafil, Macherey-Nagel, Duren, Germany) and analyzed by a chromatography system comprising the Aquity UPLC chromatograph equipped with a photodiode detector (PDA eλ Detector) (UPLC-PDA, Waters, Milford, MA, USA). The parameters of the chromatographic analysis were as follows: chromatographic column—Waters Symmetry C18 column (150 × 4.6 mm id, 5 μm); mobile phase—0.010 M sodium dihydrogen phosphate buffer adjusted with H_3_PO_4_ to pH 2.80; photodiode detection—210 nm; injection volume—3 µL; and flow rate—0.35 mL/min. For the analysts, the calibration curves showed excellent linearity with r > 0.996. Limits of detection (LOD) and quantification (LOQ) for both acids were 0.15 and 0.45 mL/L, respectively. All reagents used for analysis were of chromatographic grade and purchased from Sigma Aldrich (Darmstadt, Germany).

The total phenolic content (TPC) in fruit vinegar solutions was determined using the Folin–Ciocalteu procedure. The vinegar solution (0.1 mL) was mixed with the Folin–Ciocalteu reagent (0.25 mL) after 3 min with a Na_2_CO_3_ (Avantor, Gliwice, Poland) solution (3 mL, 10%). The absorbance at 765 nm in all solutions was measured by a Cary 300 Bio UV-Visible scanning spectrophotometer (Agilent Technologies, Santa Clara, CA, USA) after incubation for 40 min in the dark and at ambient temperature. Results were expressed as the gallic acid (Sigma Aldrich, Darmstadt, Germany) equivalent in mL of vinegar solution (µg GAeq/mL). All measurements were made in triplicate.

The total flavonoid content (TFC) in fruit vinegar solutions was performed based on the aluminum chloride colorimetric method. The vinegar solutions (0.1 mL) were mixed with methanol (0.9 mL) and AlCl_3_ (Avantor, Gliwice, Poland) solution (2 mL, 2%). The absorbance at 430 nm was read by the Cary 300 Bio UV-Visible scanning spectrophotometer (Agilent Technologies, Santa Clara, CA, USA) after incubation for 45 min in the dark and at ambient temperature. All results were expressed as µg of quercetin (Sigma Aldrich, Darmstadt, Germany), equivalent to the vinegar solution in mL (µg Qeq/mL). All measurements were made in triplicate.

### 2.3. Antioxidant Activity of Chitosan-Based Solutions

To assess the antiradical activity of fruit vinegars and chitosan solutions, the 2,2-diphenyl-1-picrylhydrazyl (DPPH) (Sigma Aldrich, Darmstadt, Germany) radical scavenging assay was employed. Briefly, each solution of fruit vinegar and chitosan solution (3 µL) was added to 297 µL of deionized water. Trolox solution (10 mg/mL in DMSO) (Sigma Aldrich, Darmstadt, Germany) was used as a reference antioxidant (positive control) and was added in an equivalent amount. A sample without solutions tested, as well as Trolox, served as a negative control (300 µL deionized water). Each sample was prepared in at least three repetitions, and the experiments were conducted twice. After vortexing (vortex IKA Works, Staufen im Breisgau, Germany), 300 µL of 0.1 mM DPPH ethanol solution was added to each sample. Following vortexing, all samples were incubated in the dark at room temperature (~24 °C) for 30 min. Subsequently, the samples were vortexed again, and the absorbance (A) of the solutions was measured at a wavelength of λ = 517 nm using a BioMateTM 160 UV-Visible spectrophotometer (Thermo Scientific, Waltham, MA, USA). The ability of all tested solutions to scavenge free radicals was calculated using the following equation:

AP (%) = [(A_nc_ − A_s_)/A_nc_] × 100%

where A_nc_ represents the absorbance value of the negative control (deionized water), and A_s_ denotes the absorbance value of the samples containing the tested solutions. The results (*n* = 10) are presented as mean values ± standard deviations (SDs).

To assess the ferrous ions (Fe^2+^) chelating activity of fruit vinegars and chitosan solutions, the ferrozine-based assay was employed. Briefly, 20 µL of 2 mM FeCl_2_ was added to a tested solution (740 µL). Ethylenediaminetetraacetic acid (EDTA) solution (10 mg/mL in deionized water) was used as a reference ferrous chelator (positive control) and was added in an equivalent amount. A sample without the tested solutions, as well as EDTA, served as a negative control. The incubation of samples (10 min) was conducted at room temperature (~24 °C). Each sample was prepared in at least three repetitions, and the experiments were conducted twice. After vortexing (vortex IKA Works, Staufen im Breisgau, Germany), 40 µL of 5 mM ferrozine in ethyl alcohol was added. Following vortexing, all samples were incubated at room temperature (~24 °C) for 10 min. Subsequently, the samples were vortexed again, and the absorbance (A) was measured at a wavelength of λ = 562 nm using a BioMateTM 160 UV-Visible spectrophotometer (Thermo Scientific, Waltham, MA, USA). The ferrous ions’ chelating activity from all tested solutions was calculated using the following formula:

Fe^2+^ chelating activity (%) = [1 − (A_s_/A_nc_)] × 100%

where A_s_ represents the absorbance value of the samples containing the tested solutions, and A_nc_ is the absorbance value of the negative control. The results (*n* = 10) are presented as mean values ± standard deviations (SDs). All reagents used in this analysis were purchased from Sigma Aldrich (Darmstadt, Germany).

### 2.4. Antimicrobial Properties

The antimicrobial effect of fruit vinegars, chitosan solutions, and chitosan films was determined using the agar diffusion method against the following bacteria strains: *Bacillus subtilis* (food isolate), *Enterococcus faecalis* (food isolate), *Escherichia coli* (ATCC 10536), *Listeria monocytogenes* (ATCC 15313), *Listeria innocua* (ATCC 19119), *Lacticaseibacillus rhamnosus* GG (ATCC 53103), *Lactiplantibacillus plantarum* 299 v (isolate from Sanprobi IBS, Poland), *Salmonella* Enteritidis (clinical isolate), *Pseudomonas aeruginosa* (ATCC 15443), and the fungal strain *Candida albicans* (stool isolate). All indicator strains were stored in a Cryobank (Bacteria storage system, MAST Diagnostica, Reinfeld, Germany) at −20 °C. Prior to the tests, the strains were defrosted and passed twice through nutrient broth (OXOID CM 0001, Basingstoke, UK) with the addition of 2% (*w*/*v*) glucose. Incubations were carried out at 35 ± 2 °C for 24 h. Muller–Hinton agar medium (OXOID CM 0337, Basingstoke, UK) was used to determine antimicrobial activity.

#### 2.4.1. Antimicrobial Activity of Fruit Vinegars and Chitosan Solutions

In the case of examining the activity of fruit vinegars and chitosan solutions on plates inoculated with standardized bacterial and yeast suspensions (10^6^ CFU/mL), wells with a diameter of 10 mm were punched. Test samples of 100 µL in volume were introduced into the wells. Plates with applied samples were then incubated at a temperature of 35 °C ± 2 °C for 48 h. After incubation, the antimicrobial activity was assessed by measuring the zones of inhibition around the wells using the Computer Scanning System (MultiScanBase v14.02).

#### 2.4.2. Antimicrobial Activity of Chitosan Films

Samples of the tested films, measuring 10 mm × 10 mm, were applied to the surfaces of plates inoculated with bacterial and yeast suspensions containing 10^6^ CFU/mL. The plates with the applied samples were then incubated at a temperature of 35 °C ± 2 °C for 48 h. After incubation, their antimicrobial activity was assessed by measuring the zones of inhibition around the applied samples. The zones of inhibition were measured using the Computer Scanning System (MultiScanBase v14.02).

### 2.5. Mechanical Properties of Chitosan Films

The strength properties of chitosan films were tested using a testing machine (Model 5965, Intron, Boston, MA, USA) following ASTMD882-12 [16] at a 100 mm/min testing speed. The average of the measurements from the ten samples is given for mechanical properties, including tensile strength (TS) and elongation at break (EB).

### 2.6. Infrared Spectroscopy

The spectra of the films were obtained using a Nicolet iS5 spectrophotometer (Thermo Fisher Scientific, Waltham, MA, USA), recording 32 scans with a spectral resolution of 4 cm^−1^ in the range of 4000–600 cm^−1^.

### 2.7. SEM Analysis

The structure of the prepared chitosan films was assessed using an Evo 40 scanning electron microscope (Zeiss, Oberkochen, Germany) operating in beam mode at 20 kV with a secondary electron detector.

### 2.8. Statistical Analysis

For statistical analyses, Tukey’s honest significant difference (HSD) test at α = 0.05 was performed using the TIBCO Software Inc. Statistica version 13.3 (Palo Alto, CA, USA).

## 3. Results and Discussion

### 3.1. Characteristics of Fruit Vinegars and Chitosan Solutions

#### 3.1.1. pH and Organic Acid Content in the Fruit Vinegars

Chitosan is a polymer easily soluble in acidic solutions below pH 6.0 [17]. The pH and the type of solvent used affect chitosan-based solutions’ bioactive properties [18] and the properties of chitosan-based films [19]. The vinegars used in the research were characterized by the following pH values: 3.6 for apple vinegar, 3.5 for blackcurrant vinegar, and 3.6 for cherry vinegar. In turn, the pH value of 3% acetic acid was 5.5, which indicates that the vinegars tested were more acidic. As shown in the literature, the pH for apple vinegar ranged from a value of 3.1 to 4.7, depending on the apple variety used in the fermentation process as well as the production method [20,21]. For cherry vinegar, the pH value oscillates between 2.7 and 3.2 [22]; these differences may also be due to the raw material used and the method of production. The obtained results, as well as data from the literature, indicate that the pH values of vinegars exceed those of 3% acetic acid, which are the most commonly used chitosan solvents in the production of chitosan films.

**Figure 1:**
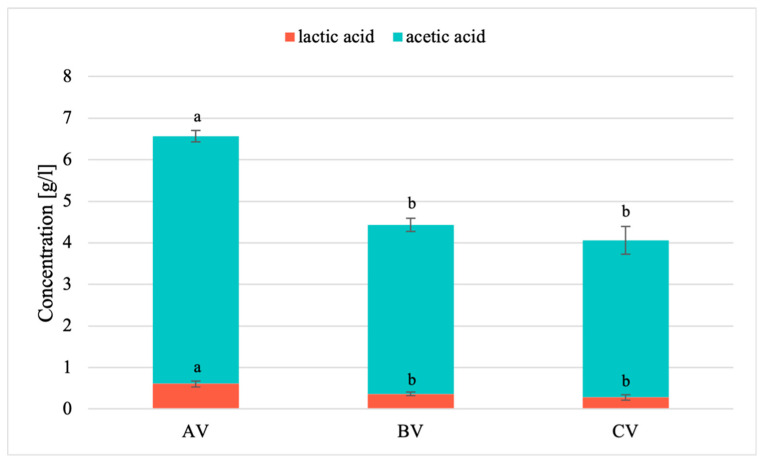
The concentration of acetic and lactic acid in vinegars: AV (apple vinegar), BV (blackcurrant vinegar), and CV (cherry vinegar). Different letters indicate samples that were significantly different (*p* < 0.05).

Fruit vinegars were characterized by a different acid content [20,21]; therefore, the concentration of acetic and lactic acid was determined in the tested vinegars, and the results are presented in Figure 1.

In all the surveyed fruit vinegars, acetic acid was dominant, and the highest content, 5.96 g/L, was determined in apple vinegar, which was statistically confirmed. However, the concentration of acetic acid determined in apple vinegar was lower than in the apple vinegars presented by Adımcılar et al. [6] and Yildiz [23]. In turn, the lactic acid content varied from 0.61 g/L (AV) to 0.28 g/L (CV). The content of lactic acid in apple vinegar determined in this study was lower than that in the study by Adımcılar et al. [6] (5.8–7.9 g/L) and higher than in the work of Yildiz [23] (0.04 g/L).

As indicated in data from the literature, the predominant acid among the acids (succinic, malic, citric, tartaric, lactic, and acetic acids) determined in fruit vinegars (apple, grape, pomegranate, and hawthorn vinegars) was acetic acid [6]. This was also confirmed in a study conducted by Yildiz [23], where of the 21 acids determined in fruit vinegars (apple, grape, rosehip, pomegranate, fig, guelder-rose, blackberry, raspberry, and blueberry), acetic acid was the dominant acid, and its content varied from 30.0 g/L in grape vinegar to 60.5 g/L in raspberry vinegar. In tests conducted on seven different vinegars from kiwi fruit apple or persimmon, among others, the most abundant organic acids were acetic acid and lactic acid, which together accounted for more than 70% of all determined acids [24]. The concentration of lactic acid and other acids present in fruit vinegars in smaller amounts, such as citric acid and malic acid, varies depending on the fermentation process [7].

One of the factors affecting the biological activity of chitosan solutions and chitosan-based films is the presence of acids such as acetic or lactic acid, among others. It is known that the type of acid used to prepare film-forming solutions is important for the properties of the obtained chitosan films [19].

#### 3.1.2. Total Phenolic and Flavonoid Content in Fruit Vinegars 

Phenolic compounds are the source of bioactive substances present in fruit vinegars, and their presence affects the antioxidant and antimicrobial properties of vinegars, among other factors. Therefore, the TPC and TFC in fruit vinegars were tested, and the results are presented in Table 2.

The tested fruit vinegars differed in total phenols and flavonoid content, depending on the type, which was confirmed by statistical analysis. The obtained results showed that the highest total phenolic compound content was found in apple vinegar, which was 360 µg of GAeq/mL, followed by blackcurrant vinegar (260 µg GAeq/mL) and cherry vinegar (210 µg GAeq/mL), which was statistically confirmed. The data from the literature showed that TPC varied depending on the type of vinegar, with cereal vinegars exhibiting higher contents than fruit vinegar [24]. The TPC of apple vinegar determined by Ren et al. [24] was 274.08 µg of gallic acid equivalent to vinegar per mL. In terms of the total flavonoid content, cherry vinegar (14.38 µg Qeq/mL) showed the highest content, followed by blackcurrant vinegar (11.03 µg Qeq/mL) and apple vinegar (6.31 µg Qeq/mL). In the case of total flavonoid content, the value obtained for apple cider vinegar was lower than the values obtained by Sidi et al. [25], who examined the flavonoid content of apple cider vinegars made from different varieties and indicated that the raw material and the production method are important factors affecting the flavonoid content of the sample.

According to the literature, the content of phenolic and flavonoid compounds may be influenced by various factors, such as the course of alcoholic or vinegar fermentation, the fermentation method and its length, as well as the type and variety of the raw material used [25]. The study showed that the total content of flavonoids and polyphenols changes over time; it was found that for the blackberry vinegar in the study for a period of 60 days, the content of flavonoids and polyphenols increased until the 15th day, and then rapidly decreased [26]. These changes were related to the depolymerization of macromolecular polyphenol or the conversion of individual polyphenol compounds by strains of lactic acid bacteria taking place during the fermentation process [26].

#### 3.1.3. Antioxidant Properties

The antioxidant properties of vinegar and chitosan solutions were assessed based on their DPPH free radical scavenging activity and ferrous ion-chelating ability.

**Figure 2:**
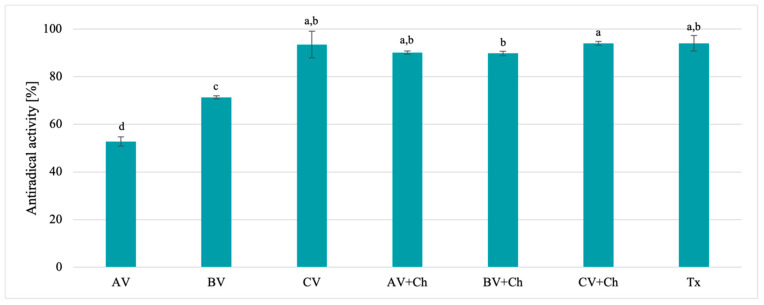
Antiradical activity of vinegar solvents of AV (apple vinegar), BV (blackcurrant vinegar), and CV (cherry vinegar), and chitosan-based solutions AV + Ch (chitosan, apple vinegar), BV + Ch (chitosan, blackcurrant vinegar), and CV + Ch (chitosan, cherry vinegar). Tx—Trolox, standard antioxidant. Different letters indicate samples that were significantly different (*p* < 0.05).

The antiradical activity outcomes obtained for natural solvents and chitosan-based solutions are presented in Figure 2. The antioxidant activity values depend on the fruits from which the vinegar was produced, which was confirmed by statistical analysis. The highest antiradical activity (93.54%) was demonstrated by cherry vinegar. This is an expected result due to the high total flavonoid content of cherry vinegar. The value obtained for samples using cherry vinegar (both the solvent itself and the solution with chitosan) was not statistically different from the commonly used standard antioxidant of Trolox. The studies on antioxidant activity revealed that chitosan film-forming solutions (AV + Ch and BV + Ch) exhibited increased antiradical activity compared to the vinegar solvents alone, which was confirmed by statistical analysis. In terms of antiradical activity, chitosan solutions produced with cherry vinegar as the solvent also demonstrated the highest efficacy (93.97%). Moreover, when compared with data from the literature, all samples showed greater antiradical activity than the film-forming chitosan solution prepared with the traditionally used solvent—acetic acid—for which antiradical activity was not noted [15]. The lower antiradical activity of chitosan solutions prepared using acetic acid compared to those using fruit vinegars was also demonstrated by Adimiclar et al. [6].

**Figure 3:**
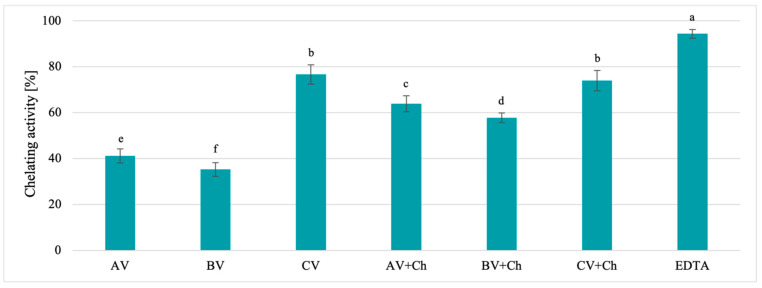
The chelating activity of vinegar solvents of AV (apple vinegar), BV (blackcurrant vinegar), and CV (cherry vinegar), and chitosan-based solutions AV + Ch (chitosan, apple vinegar), BV + Ch (chitosan, blackcurrant vinegar), and CV + Ch (chitosan, cherry vinegar). EDTA—standard chelator. Different letters indicate samples that the results were significantly different (*p* < 0.05).

The results of the examination of ferrous ions’ chelating activity obtained for natural solvents and chitosan-based solutions are presented in Figure 3.

Based on the obtained results, it can be observed that natural solvents exhibit ferrous ion-chelating activity, though this is lower than EDTA, which was confirmed by statistical analysis. The highest chelating activity was noted for a standard, which was EDTA, followed by the CV (76.64%) and CV + Ch solution (74.00%), and was statistically confirmed. The investigation of chelating activity showed that the presence of chitosan in the solution enhanced the activity of film-forming solutions based on apple vinegar from 41.13 to 63.90% and blackcurrant vinegar from 35.26 to 57.73%; no increase in activity was observed in the case of samples using cherry vinegar. Based on the obtained results, it can be observed that natural solvents exhibit antioxidant properties, which have also been confirmed in numerous studies [11,27,28,29]. Overall, it can be stated that the addition of chitosan to these solutions enhances their antioxidant properties.

The oxidation process is the main cause of food spoilage and nutrient degradation during storage. The components of packaging materials with antioxidant properties can extend shelf life and maintain food quality without the direct addition of synthetic antioxidants to food [30]. The antiradical and chelating activity of vinegar solvents, confirmed in this study, indicate their suitability for food packing materials, as they improve food safety and extend their usability for consumption over time.

#### 3.1.4. Antimicrobial Properties

Vinegar is a natural product containing many phenolic substances and organic acids, which makes it an excellent antimicrobial compound, and, as data from the literature have shown, the use of fruit vinegars as a solvent for chitosan can lead to increased antimicrobial activity [6,31].

To assess the potential use of natural vinegars as solvents for the production of chitosan-based solutions and, subsequently, for the production of films, the antimicrobial properties of the solvents and chitosan-based solutions were evaluated. The results are presented in Table 3. In addition, the statistical analysis of the results is presented in Table S1 (Supplementary Material).

The research showed that all vinegar solvents exhibited antimicrobial activity against all tested strains of pathogenic microorganisms. The tested vinegars had a stronger effect on Gram-negative bacteria than on Gram-positive bacteria. The highest activity was observed against *P. aeruginosa*, with inhibition zones exceeding 28 mm for all tested vinegars. Another strain against which high antimicrobial activity was recorded was *S.* Enteritidis, with the highest activity noted for apple vinegar, exceeding a 28 mm inhibition zone, followed by blackcurrant vinegar with a 28 mm inhibition zone, and cherry vinegar with an 18 mm inhibition zone. They also showed very high activity against *E. coli*, resulting in the appearance of inhibition zones with diameters ranging from 20 to 24 mm. Data from the literature also indicated the effectiveness of inhibiting the growth of pathogenic strains by apple vinegar, such as *Salmonella* Typhimurium, *S. aureus*, or *E. coli* [11]. Also, research conducted by Ousaaid et al. [13] indicated the effective antimicrobial activity of apple vinegars extracted from different apple varieties against the following strains: *Salmonella typhi*, *E. coli*, *Vibrio cholerae*, *C. albicans,* and *Candida tropicalis*. Against *C. albicans* yeast, the vinegars tested in this study showed moderate activity. Moreover, this activity was lower than most of the other bacteria strains.

As the obtained data show, in the case of the *P. aeruginosa* strain, all chitosan-based solutions produced using fruit vinegars retained their activity, and an inhibition zone exceeding 28 mm was noted. In the case of the *L. monocytogenes* strain, the inhibition zone increased to 16 mm for all chitosan-based solution samples compared to the results obtained for fruit vinegars alone. An increase in the activity of chitosan-based solutions was also observed compared to vinegars against the *C. albicans* strain for the BV + Ch and CV + Ch samples, as well as for the CV + Ch sample against the *S.* Enteriditis strain. As the data obtained showed, the presence of chitosan increased the activity against the selected strains.

Both fruit vinegars and the chitosan solutions made from them showed no antimicrobial activity against probiotic bacterial strains, which is favorable. This is in agreement with the expected results, as data from the literature indicate that fruit vinegars are a rich source of probiotic bacteria such as *L. plantarum* [32].

### 3.2. Characteristics of Film Samples

#### 3.2.1. Antimicrobial Properties

To assess the suitability of chitosan films produced using natural acetic solvents as potential materials for various applications, including the packaging industry, it is crucial to determine their antimicrobial activity. The results of the studies conducted are presented in Table 4. In addition, the statistical analysis of the results is presented in Table S2 (Supplementary Material).

The research demonstrated that, like the solutions (Table 3), the produced films exhibited the highest antimicrobial activity against the *P. aeruginosa* strain. The largest inhibition zone of 28 mm was recorded for the CV + ChF sample. As observed, film samples produced using cherry vinegar showed the broadest spectrum of activity against the tested pathogenic strains. None of the films tested showed activity against *E. faecalis* and *C. albicans*, despite the fact that the fruit vinegars alone had such activity. Films obtained using commonly used solvents like acetic acid did not exhibit antimicrobial activity or exhibited very poor activity; therefore, it is necessary to use additives to ensure activity [2,33], unlike films made with natural solvents such as kombucha or vinegars [3,6]. In a study conducted by Adımcılar et al. [6], antimicrobial activity was expressed as a logarithmic reduction in the number of live microorganisms; the study showed that against the *S. aureus* strain, acetic acid-based films yielded a result of about two log reductions, while for films made with pomegranate vinegar, the value increased to about three log reductions, and a similar increase in activity was observed against the *E. coli* strain. For strains such as *P. aeruginosa*, *S.* Enterica, or *E. coli*, other studies have shown that chitosan-based films made with acetic acid present no antimicrobial activity [15], while films made with bioactive solvents such as kombucha show zones of inhibition against *P. aeruginosa* in the range of 15–22 mm depending on the origin of the kombucha, which is 14–16 mm for the *S.* Enterica strain and 17 mm for the *E. coli* strain in the case of kombucha made from black tea [3]. Data from the literature confirmed the efficacy of organic acids as antimicrobial agents and indicated that individual acids differed in their antimicrobial activity against different strains. It also showed that the antimicrobial activity of films prepared with vinegar was greater than that of those with acetic acid alone. This was most likely due to the presence of organic acids as well as other bioactive compounds in their composition [34].

It is worth noting that none of the tested samples showed antimicrobial activity against the probiotic bacteria strains *L. rhamnosus* and *L. plantarum*, and similar results were noted for chitosan films where kombucha was the solvent [3]. This is a favorable result, indicating the potential use of the films for encapsulating probiotics or packaging functional food.

#### 3.2.2. Mechanical Properties

The outcomes of the mechanical tests conducted on the prepared films, evaluated in terms of tensile strength and elongation at break, are presented in Table 5.

In the case of tensile strength, values ranging from 3.97 MPa for AV + ChF to 4.93 MPa for CV + ChF were noted. The tensile strength of the chitosan film obtained by dissolving chitosan in 3% acetic acid was 38.80 MPa [15]. The obtained results showed that the use of natural solvents in film preparation led to a decrease in tensile strength for all films in comparison to films prepared with acetic acid as the solvent. The elevated tensile strength signified a high degree of ductility in films, making them easily deformable, which is a crucial characteristic of food films.

As statistical analysis indicated, the elongation at the break of chitosan films varied depending on the type of vinegar used. The chitosan films where fruit vinegars were used as the solvent were characterized by higher values of elongation at break from 48.48 to 70.58% depending on the type of vinegar used compared to chitosan film with acetic acid for which the obtained elongation at break value was 35.5% [15].

The increase in the elongation at break values observed for chitosan films obtained with fruit vinegars compared to the film with acetic acid can be attributed to the interaction of the chitosan chain with high-molecular-weight phenols present in vinegars. In addition to phenols, organic acids present in vinegars can also contribute to improving the elasticity of the film. Chitosan films based on malic, lactic, or citric acid exhibited greater elasticity than chitosan films with acetic acid [35]. The reaction between the solvent and chitosan, resulting from electrostatic interactions, hydrogen bonding, or hydrophobic interactions, influenced the structure and properties of chitosan films [36]. Numerous studies emphasized the substantial impact of acids on chitosan through electrostatic interactions [19], reaffirming that many applications of chitosan are based on its cationic nature. The volume of acids as counter ions was expected to influence the ionic interactions between amino and carboxyl groups, thus affecting the properties of chitosan. Additionally, hydrophobic interactions between chitosan and carboxylic acids could occur, influencing the sorption of carboxylic acids on chitosan [37]. This was confirmed by the conducted test results, which demonstrated the presence of acetic and lactic acids in the tested vinegars (Figure 1).

#### 3.2.3. Attenuated Total Reflectance Fourier Transform Infrared Spectroscopy (ATR-FTIR)

**Figure 4:**
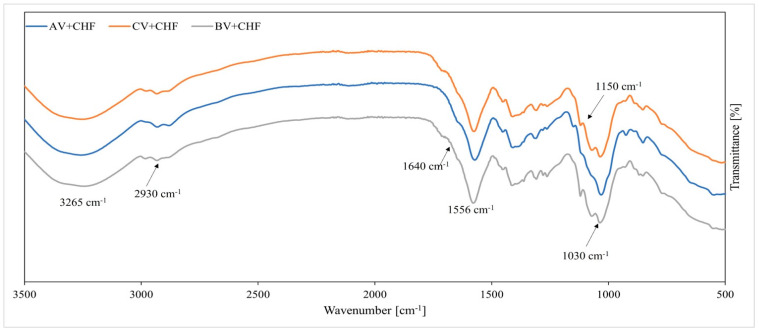
FTIR spectra of the prepared chitosan-based films: AV + Ch (chitosan, apple vinegar), BV + Ch (chitosan, blackcurrant vinegar), and CV + Ch (chitosan, cherry vinegar).

The chitosan-based film structures were analyzed using attenuated total reflectance Fourier transform infrared spectroscopy. Figure 4 displays the spectra of all the examined chitosan-based films.

The prepared materials exhibited the characteristic peaks of chitosan within their structures. The bands observed at 3265 cm^−1^ correspond to N-H stretching vibrations overlapping with O-H stretching, and the absorption band at 2930 cm^−1^ can be attributed to C-H asymmetric stretching vibrations [6,38]. The bands at 1640 and 1556 cm^−1^ can be assigned to amide I (C=O stretching), amide II (N-H bending), and amide III (C-N stretching), respectively [6,39,40]. The bands at 1150 and 1030 cm^−1^ can be associated with the symmetric stretching of the C-O-C bond and C-O stretching vibrations, respectively [38,40]. The obtained data are consistent with data from the literature, which showed that the use of fruit vinegars does not indicate the presence of significant changes in FTIR spectra [6].

#### 3.2.4. Scanning Electron Microscopy (SEM)

**Figure 5:**
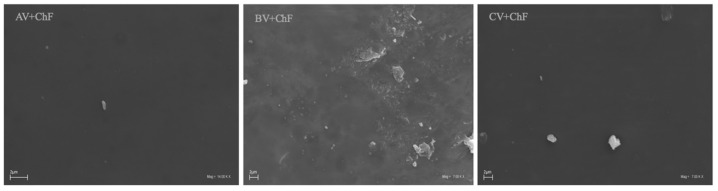
SEM micrographs for prepared films: AV + ChF (chitosan, apple vinegar), BV + ChF (chitosan, blackcurrant vinegar), and CV + ChF (chitosan, cherry vinegar). The scale bar is 2 µm.

In this study, scanning electron microscopy (SEM) was employed to observe the surface morphology of the tested samples, and the results in form of micrographs are presented in Figure 5.

The SEM analysis of the structure and morphology of the resulting films revealed smooth surfaces in all the chitosan films. This observation indicated well-dispersed chitosan in the natural vinegars used, which is a highly desirable quality for the formation of films intended for various applications.

## 4. Conclusions

In this study, three types of fruit vinegars were used both as chitosan solvents and as a source of biologically active compounds for the production of chitosan-based films. Fruit vinegars showed antioxidant and antimicrobial activity, which is associated with the presence of organic acids and phenolic compounds. Both fruit vinegars and film-forming chitosan solutions demonstrated antimicrobial properties against the tested microbial strains. The antimicrobial effectiveness was maintained in chitosan-based films produced with cherry vinegar, which exhibited the broadest spectrum of bacterial activity. None of the tested samples showed antimicrobial activity against the probiotic bacteria strains *L. rhamnosus* and *L. plantarum*, which is a favorable result. The tests also revealed that the vinegar type influenced the mechanical properties of the films, such as elongation at break. SEM studies showed that the obtained films have a smooth surface, which indicates the good dispersion of chitosan in the natural vinegar solvent. The research results obtained showed that depending on the solvent used, films with different mechanical properties and different biological activity can be obtained, which allows for the appropriate selection of the solvent for a given application and may be an important aspect, among others, in the selection of the best packaging for a given type of food. Thus, natural fruit vinegars offer a promising environmentally friendly alternative to conventional solvents for the production of chitosan-based films with improved functional properties.

## Figures and Tables

**Table 1:** Sample composition and abbreviations.

Solvent	Abbreviation	
	Chitosan Solution	Chitosan Film
Apple vinegar (AV)	AV + Ch	AV + ChF
Blackcurrant vinegar (BV)	BV + Ch	BV + ChF
Cherry vinegar (CV)	CV + Ch	CV + ChF

**Table 2:** Total phenolic compounds and flavonoid content.

Symbol	TPC (µg GAeq/mL)	TFC (µg Qeq/mL)
AV	360 ^a^ ± 28	6.31 ^c^ ± 0.14
BV	260 ^b^ ± 18	11.03 ^b^ ± 0.11
CV	210 ^c^ ± 13	14.38 ^a^ ± 0.08

Different letters indicate samples that were significantly different (*p* < 0.05).

**Table 3:** Antimicrobial properties of vinegar of AV (apple vinegar), BV (blackcurrant vinegar), and CV (cherry vinegar), and chitosan-based solutions AV + Ch (chitosan, apple vinegar), BV + Ch (chitosan, blackcurrant vinegar), and CV + Ch (chitosan, cherry vinegar).

Indicator Strain										
	*B. subtilis*	*E. faecalis*	*L. monocytogenes*	*L. innocua*	*E. coli*	*P. aeruginosa*	*S. enteritidis*	*L. rhamnosus*	*L. plantarum*	*C. albicans*
Sample	Inhibition zone [mm]									
AV	20	22	14	15	24	>28	>28	0	0	13
BV	18	23	15	18	24	>28	28	0	0	11
CV	18	18	15	16	20	>28	18	0	0	11
AV + Ch	14	0	16	18	14	>28	23	0	0	11
BV + Ch	14	0	16	16	14	>28	18	0	0	12
CV + Ch	14	0	16	16	14	>28	22	0	0	12

Diameter of the resulting zones of translucence: 5–10 mm—weak activity; 11–14 mm—moderate activity; and >14 mm—strong activity.

**Table 4:** Antimicrobial properties of films AV + ChF (chitosan, apple vinegar), BV + ChF (chitosan, blackcurrant vinegar), and CV + ChF (chitosan, cherry vinegar).

Indicator Strain										
	*B. subtilis*	*E. faecalis*	*L. monocytogenes*	*L. innocua*	*E. coli*	*P. aeruginosa*	*S. enteritidis*	*C. albicans*	*L. rhamnosus*	*L. plantarum*
Sample	Inhibition zone [mm]									
AV + ChF	0	0	0	0	0	19	0	0	0	0
BV + ChF	0	0	0	0	11.5	19	11	0	0	0
CV + ChF	20	0	19	20	24	28	11	0	0	0

Diameter of the resulting zones of translucence: 5–10 mm—weak activity; 11–14 mm—moderate activity; and >14 mm—strong activity.

**Table 5:** Tensile strength (TS) and elongation at break (EB) for the prepared films.

Symbol	TS (MPa)	EB (%)
AV + ChF	3.97 ^b^ ± 0.18	48.48 ^c^ ± 1.06
BV + ChF	3.82 ^b^ ± 0.33	70.58 ^a^ ± 1.19
CV + ChF	4.93 ^a^ ± 0.63	51.07 ^b^ ± 0.54

Different letters indicate samples that were significantly different (*p* < 0.05).

## Data Availability

The original contributions presented in this study are included in the article/Supplementary Material. Further inquiries can be directed to the corresponding author.

## Supplementary Materials

The following supporting information can be downloaded at https://www.mdpi.com/article/10.3390/polym17010011/s1. Table S1: Antimicrobial properties of vinegar: AV (apple vinegar), BV (blackcurrant vinegar), CV (cherry vinegar), and chitosan-based solutions AV + Ch (chitosan, apple vinegar), BV + Ch (chitosan, blackcurrant vinegar), and CV + Ch (chitosan, cherry vinegar); Table S2: Antimicrobial properties of films AV + ChF (chitosan, apple vinegar), BV + ChF (chitosan, blackcurrant vinegar), and CV + ChF (chitosan, cherry vinegar).
