# Supplementary material for: Fruit Vinegars as Natural and Bioactive Chitosan Solvents in the Production of Chitosan-Based Films
Source: Polymers (Basel). 2024 Dec 25;17(1):11. doi: 10.3390/polym17010011 (PMC11723316; doi:10.3390/polym17010011)
Supplement: Supplementary file 1 [file polymers-17-00011-s001.zip › polymers-3332163-supplementary.pdf]

Table S1. Antimicrobial properties of vinegar: AV (apple vinegar), BV (blackcurrant vinegar), CV (cherry vinegar) and chitosan-based solutions AV+Ch (chitosan, apple vinegar), BV+Ch (chitosan, blackcurrant vinegar), CV+Ch (chitosan, cherry vinegar).

	Indicator strain									
	B. subtilis	*E. faecalis*	*L. monocytogenes*	*L. innocua*	*E. coli*	*P. aeruginosa*	*S.* Enteritidis	*L. rhamnosus*	*L. plantarum*	*C. albicans*
**Sample**	**Inhibition zone [mm]**									
**AV**	$20^{a}\pm1.5$	$22^{a}\pm1.5$	$14^{a}\pm1$	$15^{a}\pm1$	$24^{a}\pm2$	>28	>28	0	0	$13^{a}\pm1$
**BV**	$18^{a}\pm1.5$	$23^{a}\pm2$	$15^{a}\pm1$	$18^{a}\pm1.5$	$24^{a}\pm2$	>28	$28^{a}\pm2$	0	0	$11^{a}\pm1$
**CV**	$18^{a}\pm1.5$	$18^{b}\pm1.5$	$15^{a}\pm1$	$16^{a}\pm1$	$20^{a}\pm1.5$	>28	$18^{c}\pm1.5$	0	0	$11^{a}\pm1$
**AV+Ch**	$14^{b}\pm1$	0^{c}	$16^{a}\pm1$	$18^{a}\pm1.5$	$14^{b}\pm1$	>28	$23^{b}\pm2$	0	0	$11^{a}\pm1$
**BV+Ch**	$14^{b}\pm1$	0^{c}	$16^{a}\pm1$	$16^{a}\pm1$	$14^{b}\pm1$	>28	$18^{c}\pm1.2$	0	0	$12^{a}\pm1$
**CV+Ch**	$14^{b}\pm1$	0^{c}	$16^{a}\pm1$	$16^{a}\pm1$	$14^{b}\pm1$	>28	$22^{b,c}\pm1.5$	0	0	$12^{a}\pm1$

Diameter of the resulting zones of translucence: 5-10 mm – weak activity; 11-14 mm – moderate activity; >14 mm – strong activity. Different letters indicate samples that were significantly different ($p < 0.05$). Results for the inhibition zone above 28 mm and for 0 mm were not subject to statistical evaluation.

Table S2. Antimicrobial properties of films AV+ChF (chitosan, apple vinegar), BV+ChF (chitosan, blackcurrant vinegar), CV+ChF (chitosan, cherry vinegar).

	Indicator strain									
	B. subtilis	*E. faecalis*	*L. monocytogenes*	*L. innocua*	*E. coli*	*P. aeruginosa*	*S.* Enteritidis	*C. albicans*	*L. rhamnosus*	*L. plantarum*
**Sample**	**Inhibition zone [mm]**									
**AV+ChF**	0	0	0	0	0	$19^{b}\pm1.5$	0	0	0	0
**BV+ChF**	0	0	0	0	$11.5^{b}\pm1$	$19^{b}\pm1.5$	$11^{a}\pm1$	0	0	0
**CV+ChF**	20	0	19	20	$24^{a}\pm2$	$28^{a}\pm2$	$11^{a}\pm1$	0	0	0

Diameter of the resulting zones of translucence: 5-10 mm - weak activity; 11-14 mm - moderate activity; >14 mm - strong activity. Different letters indicate samples that were significantly different ($p < 0.05$). Results for the inhibition zone above 28 mm and for 0 mm were not subject to statistical evaluation.
